# Ethical Issues Regarding Nonsubjective Psychedelics as Standard of Care

**DOI:** 10.1017/S096318012200007X

**Published:** 2022-10

**Authors:** David B. Yaden, Brian D. Earp, Roland R. Griffiths

**Affiliations:** 1 Department of Psychiatry and Behavioral Sciences, Johns Hopkins University School of Medicine, Baltimore, Maryland 21224, USA; 2 The Yale-Hastings Program in Ethics and Health Policy, Yale University and the Hastings Center, New Haven, Connecticut 06511, USA; 3 Uehiro Centre for Practical Ethics, University of Oxford, Oxford OX1 1PT, UK; 4 Department of Neuroscience, Johns Hopkins University, Baltimore, Maryland 21224, USA

**Keywords:** psychedelics, hallucinogens, bioethics, psychiatry

## Abstract

Evidence suggests that psychedelics bring about their therapeutic outcomes in part through the subjective or qualitative effects they engender and how the individual interprets the resulting experiences. However, psychedelics are contraindicated for individuals who have been diagnosed with certain mental illnesses, on the grounds that these subjective effects may be disturbing or otherwise counter-therapeutic. Substantial resources are therefore currently being devoted to creating psychedelic substances that produce many of the same biological changes as psychedelics, but without their characteristic subjective effects. In this article, we consider ethical issues arising from the prospect of such potential “nonsubjective” psychedelics. We are broadly supportive of efforts to produce such substances for both scientific and clinical reasons. However, we argue that such nonsubjective psychedelics should be reserved for those special cases in which the subjective effects of psychedelics are specifically contraindicated, whereas classic psychedelics that affect subjective experience should be considered the default and standard of care. After reviewing evidence regarding the subjective effects of psychedelics, we raise a number of ethical concerns around the prospect of withholding such typically positive, meaningful, and therapeutic experiences from most patients.

## Introduction

Psychedelics reliably produce substantially altered states of consciousness—but how do these subjectively experienced effects relate to the therapeutic effects of psychedelics demonstrated in recent research? Could a psychedelic be created that has no subjective effects (i.e., a “nonsubjective” psychedelic) but identical therapeutic impacts? The prospect of such nonsubjective psychedelics is scientifically and clinically interesting but raises several bioethical considerations.

Two distinct views have emerged regarding the question of whether it is possible for a nonsubjective psychedelic to have the same therapeutic impact as currently available psychedelic substances, with their characteristic effects on subjective experience (i.e., altered states of consciousness). On the one hand, David Olson^1^ has suggested that the subjective effects of psychedelics are likely “unnecessary for their therapeutic effects.” According to this view, currently measurable biological processes which have no subjective correlates (e.g., neurogenesis and neuroplasticity) can entirely account for the benefits of psychedelics and are all that are needed to achieve their therapeutic impact (e.g., reducing depression or treating substance use disorders as well as increasing well-being—described below).

On the other hand, some researchers have suggested that the subjective effects of psychedelics may be “necessary for their *full and enduring* therapeutic effects.”^2^ According to this contrary view, certain cognitive and affective shifts from the subjective effects of psychedelics account for a large degree of the size and longevity of their beneficial effects. Such changes may involve, for example, explicit adoption of alternative conceptual frameworks through which to view and interpret one’s experiences, motivations, and social relationships.^3^ While we can imagine nonsubjective psychedelics having some (kinds of) therapeutic impact, we believe it is highly likely that such impact will be less strong and less enduring than what can be achieved through the experience of, and conscious engagement with, the subjective effects of psychedelics.

Data from ongoing preclinical and clinical studies may help adjudicate between these two views. For example, studies using experimental methods such as the controlled manipulation of exposure to anesthesia could provide relevant evidence. In what David B. Yaden and Roland R. Griffiths (see note 2) describe as a “critical test,” psychedelics would be introduced during deep anesthesia (expected to eliminate any subjective effects of the psychedelics due to the anesthesia-induced suppression of conscious awareness) in one experimental condition and during ordinary waking consciousness in another condition. If the former (anesthetized) mode of administration resulted in equivalent and equally enduring therapeutic effects as psychedelics administered without anesthesia, then this would provide evidence in favor of Olson’s^4^ view. Another way to test the relative strength of the competing views would be to create psychedelics that act in a nearly identical biological manner but which do not produce subjective effects, as is currently being actively explored.

Psychedelics that produce therapeutic benefits, but which do not produce a substantially altered state of consciousness, have not yet been created. However, a large amount of funding from military and industry sources has been allocated in order to pursue the possibility of psychedelics or psychedelic-like substances of this kind effects.^5^ The rationale for such efforts has been,^6^ broadly: (1) the subjective effects may not have any causal role in the therapeutic effects (as mentioned, this issue has not been scientifically settled), (2) the subjective effects of psychedelics could be harmful to some people (e.g., those with a personal or family history of psychotic disorders), for who experiencing the subjective effects of psychedelics is indeed contraindicated,^7^ and (3) the cost of treatment could be reduced as there might be less of a need for active clinical support during the experience of acute subjective effects (as these may not need to occur). There is no doubt that the discovery of such substances would be valuable scientifically (e.g., to address the first point) and in some cases clinically (e.g., to address the second and third points).

The issue at stake in the present article is not whether such substances should be created, as we agree they would have considerable value. Instead, the ethical issues we are concerned with arising when considering whether such possible nonsubjective psychedelics should be the *default* treatment option. That is, granting the possibility that nonsubjective psychedelics can be created, what should be the standard of care for most patients?

## Psychedelics and Treatment Potential

Substances considered classic psychedelics include psilocybin (derived from the psilocybe mushroom), lysergic acid diethylamide (LSD), *N*,*N*-dimethyltryptamine (DMT), and mescaline (3,4,5-trimethoxyphenethylamine).^8^ With the exception of LSD, which was produced synthetically in 1938 in Switzerland, the naturally occurring psychedelic substances have been used in communal spiritual settings and religious rituals across a number of cultures for generations.^9^ In the 20th century, psychedelics have also been referred to as “hallucinogens,” “entheogens,” and “psychomimetics” due to the nature of the substantially altered states of consciousness they elicit.^10^

In the past two decades, clinical trials on psilocybin have demonstrated a significant potential to increase well-being,^11^ ^,^^12^ ^,^^13^ as well as decrease anxiety,^14^ ^,^^15^ decrease depression,^16^ ^,^^17^ ^,^^18^ and treat substance use disorders such as alcohol use disorder and tobacco use disorder.^19^ ^,^^20^ Psilocybin has been granted the “breakthrough therapy” designation by the United States Food and Drug Administration (FDA) and is currently in Phase 2b trials for the treatment of Major Depressive Disorder and is being explored for its treatment potential across a range of other mental disorders.

## The Possibility and Scientific Usefulness of Psychedelics with No Subjective Effects

As Olson^21^ argues, it may be possible to develop substances that produce equivalent therapeutic benefits to psychedelics without their subjective effects. If so, this would likely be due to several basic neurobiological processes associated with psychedelics that have been elucidated. Psychedelics, in both in vivo and in vitro studies, have been measured and shown to produce neurogenesis^22^ ^,^^23^; they are also known to foster neural plasticity.^24^ The prospect of creating chemical substances that are capable of promoting processes such as neurogenesis and neural plasticity without triggering subjectively experienced altered states of consciousness is actively being pursued, as noted, through many millions of dollars of funding from the Defense Advanced Research Projects Agency (DARPA) and industry sources.

The motivation behind efforts to produce nonsubjective psychedelics appears, as mentioned, to be two-fold—scientific and clinical. In addition to the aforementioned scientific question regarding whether the altered state of consciousness associated with psychedelics is causally necessary to produce the therapeutic effects of these substances (but there is a need to carefully qualify issues related to studying “consciousness” with psychedelics),^25^ there are also questions around basic neuropsychopharmacology at stake that could be better understood through this process. Clinically, the subjective effects of psychedelics may prevent certain groups of people from receiving them—such as those with a history (including family history) of psychotic disorders. Currently, this is considered a risk factor for negative outcomes and is generally an exclusion criterion for both research and therapy,^26^ although it is not yet established whether potential nonsubjective psychedelics would be safe for these patient populations. In any case, we want to reiterate our support efforts to create such substances.

There are, however, important downsides to consider regarding using these potential substances as the default option or the standard of care.

## The Subjective Effects of Psychedelics

The nature of the subjective effects of psychedelics—which so many resources are currently being devoted to removing—has been variously characterized, but the field is working toward a convergence. The effects of psychedelics involve perceptual changes such as seeing patterns and vivid inner imagery, intense positive and negative emotions, as well as feelings of connection and ego-dissolution.^27^ ^,^^28^

The acute subjective effects of psychedelics can be psychologically challenging (colloquially described as “bad trips”). The unusual and sometimes confusing nature of the subjective effects can cause anxiety and even panic in some people. A survey of recreational psychedelic users who had “challenging” experiences on psilocybin (i.e., not a representative sample) found that 39% of the respondents rated it among the most challenging experiences in their life (note: this is not always interpreted as negative or regrettable; “challenging” experiences are often those from which a person learns the most), but, more ominously, 11% reported putting themselves or others at some risk, 2.7% required medical attention, and 7.6% subsequently sought treatment for enduring psychological symptoms that they attributed to the experience.^29^ In two psychedelic administration studies in psychedelic naïve participants, strong or extreme ratings of fear occurred in 31%^30^ and fear and other related states were reported in 39% of participants^31^ at some time during sessions, with sustained anxiety or unpleasant psychological struggle during sessions occurring in 11% of participants.^32^ Again, these participants did not uniformly consider the experiences of fear or anxiety as rendering the whole experience undesirable; rather, such negative emotions were often interpreted as meaningful in themselves and/or accompanied by positive emotions or feelings of growth.^33^

In terms of lasting negative impacts of psychedelics, postsession negative symptoms occurred in 0.9% of 110 participants in drug administration studies at the Vollenweider laboratory in Switzerland (see note 26) and, likewise in 0.9% of 250 participants in studies at Johns Hopkins.^34^

Despite these challenging experiences during sessions and some risk of lingering negative effects in terms of psychological stress, psilocybin has been rated as among the safest of all so-called “recreational” drugs in terms of risk of harm to oneself or others.^35^ Moreover, classic psychedelics show a positive safety profile (including with respect to neurotoxicity or potential for addiction) when compared with many commonly prescribed drugs used for medication.^36^

Crucially for our discussion here, in addition to the fact that many temporally limited negative emotions (e.g., fear and anxiety) are subsequently interpreted as meaningful or instrumental for achieving personal insights or growth, the acute subjective effects of psychedelics when administered under supportive conditions are rated as producing substantial increases in a deeply felt positive mood (e.g., feelings of peace, tranquility, and joy).^37^ ^,^^38^ ^,^^39^ ^,^^40^

In terms of persisting positive effects, there are a number of well-being related outcomes that are may be orthogonal to therapeutic effects. In a randomized cross-over trial comparing psilocybin to methylphenidate, participants reported much larger increases in positive attitudes about life and/or self, positive mood changes, altruistic/positive social effects, positive behavior change, and well-being 2 months after a psilocybin session than after a methylphenidate session.^41^ Another study involving low and high doses of psilocybin^42^ found that 1 month after high dose psilocybin sessions, 94% of participants endorsed an increased sense of well-being and 89% endorsed positive behavior change. One finding is worth special mention in this context—the well-replicated finding in at least four clinical trials that *most participants (mean 76%, range 58–94%) rate their psychedelic experiences among the most meaningful experiences of their entire lives 6–14 months after their last session.* ^43^ ^,^^44^ ^,^^45^ ^,^^46^

Despite these promising findings, we caution that more research is needed, especially better controlled and larger trials before firm conclusions can be drawn regarding these seemingly highly positive effects.^47^ With these caveats in place, we believe that the current evidence suggests that the subjective effects of psychedelics pose some risk of inducing a challenging and stress-inducing experience that may in some cases have lingering negative psychological consequences, but that the vast majority of participants report highly beneficial effects that are in many cases considered extraordinarily meaningful.

## Risks and Benefits of the Subjective Effects of Psychedelics

If we grant the hypothetical case that nonsubjective psychedelics were created that were shown to be *equally effective* at reducing identified mental disorders, then should subjective or nonsubjective psychedelics be the default treatment?

Olson^48^ has argued that nonsubjective psychedelics should be the default treatment for several reasons. He emphasizes the risks associated with subjective effects of psychedelics, particularly for some patient populations—but also for people in general. As we have acknowledged, there are indeed risks from psychedelic experiences to some individuals, particularly those who have a personal or family history with psychotic disorders. There is no question that psychedelic experiences can be challenging or negative, especially in some patient populations. We have no disagreement here and would indeed welcome nonsubjective psychedelics for this clinical application.

Another reason raised by Olson^49^ is the clinical costs associated with administering psychedelics in clinical settings. Typically, clinicians must spend several hours with patients. Instead, Olson argues, participants under the influence of the postulated nonsubjective psychedelics would need no supervision. This would cut healthcare costs, or so the argument goes. We agree that it is possible that nonsubjective psychedelics could reduce economic costs—but at what human costs?

Already, there is a broad movement in medical ethics critiquing bioreductive approaches to treatment, where mental health is conceived of in terms of brain states or chemical processes alone rather than (also) in terms of a person’s lived experiences in sociorelational context, and the meanings they give to those experiences as part of a life narrative.^50^ To be a person, much less a person in good mental health, is to experience and interpret the world in a certain way (or range of ways); it is not simply to be a bearer of nonpathological brain states. Accordingly, clinician-supported, psychedelic-assisted therapy involving an interpersonal process of interpretation, meaning-making, and integration, is considered the gold standard for treatments involving psychedelics.^51^ Thus, even if it is possible that nonsubjective psychedelics could bring about equivalent treatment effects in terms of measurable decrements in clinical symptoms, it is highly unlikely that they would also replicate the less-tangible, but perhaps no less important, effects on well-being derived from the human-to-human therapeutic encounter and associated sense-making of the narrative content of drug-induced subjective experiences or altered states of consciousness.

## Ethical Considerations

In the domain of morality, there is a helpful distinction between negative morality and positive morality. Negative morality describes moral rules or laws that are widely socially recognized and interpreted as binding, which prevent us from taking certain actions, or tell us what we ought *not* to do. Positive morality, on the other hand, asks what we ought *to* do. This distinction is instructive when applied to the questions of subjective or nonsubjective psychedelics. If, throughout the course of effective treatments, we can help foster one of the most positive and meaningful experiences of a patient’s life without undue risk, then, from the perspective of positive morality, we may be under some obligation to provide such an experience.

Such an approach is consistent with long-standing operationalizations of health, such as that adopted by the World Health Organization in 1948. According to the WHO,^52^ “Health is a state of complete physical, mental and social well-being and not merely the absence of disease or infirmity.” It is uncontroversial that a major purpose of medicine is to improve the health of the population. In this regard, a negative approach to morality might instruct us to avoid taking actions that are likely to cause disease or infirmity. At best, it requires us to apply the tools of medicine to treat existing problems (since available treatments ought not to be withheld). However, a positive approach to morality might also include a duty to promote, in a more holistic fashion, the physical, mental, and social well-being of patients within our care. Stated differently, as bioethicists have long argued, the moral aims of medicine encompass more than simply “do no harm” (i.e., the principle of nonmaleficence); rather, clinicians and other healthcare providers have a positive ethical duty of beneficence (i.e., to do good).^53^

Consider the hypothetical availability of two otherwise equivalent chemical substances:

1. One substance with the desired treatment effect vis-à-vis a circumscribed mental disorder and no adverse side-effects stemming from taking the drug
2. Another substance with the same desired treatment effects, a small risk of adverse side-effects stemming from the subjective experience of taking the drug, but also a high probability of a profoundly positive subjective experience as well as other persisting positive effects

It may be difficult to assign precise weights to the benefits and risks of each intervention; however, a broad analysis can still be given. The expected value or disvalue of an intervention is a product of both the likelihood of good or bad outcomes and the magnitude or degree of each. Given the available data regarding relatively low likelihood of adverse experiences (especially lasting negative experiences of any great magnitude) as well as a very high likelihood of positive experiences that are weighted as being of exceptional magnitude, we believe that consequentialist calculations would pick the second intervention as by far the more beneficent. To illustrate, less than 1% of participants report lingering negative effects (and of those, only a minority require therapeutic support), whereas about three-quarters of participants report the experience to be, as noted, among the most meaningful of their entire lives.

Another major principle of bioethics is the need to ground healthcare decisions in a stance of respect for persons, often operationalized in terms of valuing the autonomy of the individual. Autonomy means allowing individuals to choose those ends which they themselves value, so long as others are not unduly harmed (or disrespected) in the pursuit of those ends. Almost everyone values well-being (which is typically enhanced by the subjective experience of psychedelics taken under the right conditions), but this is not the only valuable end that people may pursue. Rather, having a meaningful life is also highly valued by most people, and it is notable that the typical subjective effects of psychedelics are often characterized in terms of *meaningfulness*, not simply increased positive mood or even general well-being.^54^ Such outcomes would presumably not be associated with nonsubjective psychedelics.

## Conclusion

Given the choice, many individuals would likely autonomously choose to undergo a treatment with a high likelihood of being beneficial as well as capturing other valuable ends, such as the meaningfulness of the experience. In addition to beneficence, therefore, there are reasons rooted in autonomy and respect for persons at least to *offer* classic psychedelics, with their attendant subjective effects, as the default treatment option and standard of care for those who do not have specific contraindications, while also allowing patients to choose the nonsubjective route if that is what would make them more comfortable. Overall, we support scientific efforts to create nonsubjective psychedelics, but we hope to ensure that the subjective effects of classic psychedelics remain an option for those who are willing and able, as many clinical trial participants report that this experience ranks among the most positive and meaningful of their lives.

## Notes

1.

Olson DE . The subjective effects of psychedelics may not be necessary for their enduring therapeutic effects. ACS Pharmacology & Translational Science 2020;4(2):563–73386121810.1021/acsptsci.0c00192PMC8033607.

2.

Yaden DB , Griffiths RR . The subjective effects of psychedelics are necessary for their enduring therapeutic effects. ACS Pharmacology & Translational Science 2020;4(2):568–23386121910.1021/acsptsci.0c00194PMC8033615.

3.

Earp BD , Savulescu J. Psychedelic relationship enhancement: Précis of love drugs. Philosophy and Public Issues 2020;10(3):3–28 .

4. See note 1, Olson 2020, at 563–7.

5. DARPA. *Structure-Guided Drug Design Could Yield Fast-Acting Remedies for Complex Neuropsychiatric Conditions*; available at https://www.darpa.mil/news-events/2019-09-11 (last accessed 2 Feb 2022).

6. See note 1, Olson 2020, at 563–7.

7.

Johnson MW , Richards WA , Griffiths RR . Human hallucinogen research: Guidelines for safety. Journal of Psychopharmacology 2008;22(6):603–201859373410.1177/0269881108093587PMC3056407.

8.

Nichols DE . Psychedelics. Pharmacological Reviews 2016;68(2):264–355 2684180010.1124/pr.115.011478PMC4813425.

9.

Schultes RE . Hallucinogens of plant origin: Interdisciplinary studies of plants sacred in primitive cultures yield results of academic and practical interest. Science 1969;163(3864):245–54488361610.1126/science.163.3864.245.

10. See note 8, Nichols 2016, at 264–355.

11.

Griffiths RR , Richards WA , McCann U , Jesse R. Psilocybin can occasion mystical-type experiences having substantial and sustained personal meaning and spiritual significance. Psychopharmacology 2006;187(3):268–831682640010.1007/s00213-006-0457-5.

12.

Griffiths RR , Johnson MW , Richards WA , Richards BD , McCann U , Jesse R. Psilocybin occasioned mystical-type experiences: Immediate and persisting dose-related effects. Psychopharmacology 2011;218(4):649–652167415110.1007/s00213-011-2358-5PMC3308357.

13.

Griffiths RR , Johnson MW , Richards WA , Richards BD , Jesse R , MacLean KA , et al. Psilocybin-occasioned mystical-type experience in combination with meditation and other spiritual practices produces enduring positive changes in psychological functioning and in trait measures of prosocial attitudes and behaviors. Journal of Psychopharmacology 2018;32(1):49–69 2902086110.1177/0269881117731279PMC5772431.

14.

Griffiths RR , Johnson MW , Carducci MA , Umbricht A , Richards WA , Richards BD , et al. Psilocybin produces substantial and sustained decreases in depression and anxiety in patients with life-threatening cancer: A randomized double-blind trial. Journal of Psychopharmacology 2016;30(12):1181–972790916510.1177/0269881116675513PMC5367557.

15.

Ross S , Bossis A , Guss J , Agin-Liebes G , Malone T , Cohen B , et al. Rapid and sustained symptom reduction following psilocybin treatment for anxiety and depression in patients with life-threatening cancer: A randomized controlled trial. Journal of Psychopharmacology 2016;30(12):1165–80.2790916410.1177/0269881116675512PMC5367551

16.

Carhart-Harris RL , Bolstridge M , Day CM , Rucker J , Watts R , Erritzoe DE , et al. Psilocybin with psychological support for treatment-resistant depression: Six-month follow-up. Psychopharmacology 2018;235(2):399–408 2911921710.1007/s00213-017-4771-xPMC5813086.

17.

Carhart-Harris R , Giribaldi B , Watts R , Baker-Jones M , Murphy-Beiner A , Murphy R , et al. Trial of psilocybin versus escitalopram for depression. New England Journal of Medicine 2021;384(15):1402–113385278010.1056/NEJMoa2032994.

18.

Davis AK , Barrett FS , May DG , Cosimano MP , Sepeda ND , Johnson MW , et al. Effects of psilocybin-assisted therapy on major depressive disorder: A randomized clinical trial. JAMA Psychiatry 2021;78(5):481–93314666710.1001/jamapsychiatry.2020.3285PMC7643046.

19.

Bogenschutz MP , Forcehimes AA , Pommy JA , Wilcox CE , Barbosa PC , Strassman RJ . Psilocybin-assisted treatment for alcohol dependence: A proof-of-concept study. Journal of Psychopharmacology 2015;29(3):289–992558639610.1177/0269881114565144.

20.

Johnson MW , Garcia-Romeu A , Cosimano MP , Griffiths RR . Pilot study of the 5-HT2AR agonist psilocybin in the treatment of tobacco addiction. Journal of Psychopharmacology 2014;28(11):983–922521399610.1177/0269881114548296PMC4286320.

21. See note 1, Olson 2020, at 563–7.

22.

Ly C , Greb AC , Cameron LP , Wong JM , Barragan EV , Wilson PC , et al. Psychedelics promote structural and functional neural plasticity. Cell Reports 2018;23(11):3170–822989839010.1016/j.celrep.2018.05.022PMC6082376.

23.

Shao LX , Liao C , Gregg I , Davoudian PA , Savalia NK , Delagarza K , et al. Psilocybin induces rapid and persistent growth of dendritic spines in frontal cortex in vivo. Neuron 2021;109(16):2535–44.e43422895910.1016/j.neuron.2021.06.008PMC8376772.

24.

Olson DE . Psychoplastogens: A promising class of plasticity-promoting neurotherapeutics. Journal of Experimental Neuroscience 2018;12:117906951880050810.1177/1179069518800508PMC614901630262987.

25.

Yaden DB , Johnson MW , Griffiths RR , Doss M , Garcia-Romeu A , Nayak S , et al. Psychedelics and consciousness: Distinctions, demarcations, and opportunities. International Journal of Neuropsychopharmacology 2021;24(8):615–233398765210.1093/ijnp/pyab026PMC8378075.

26.

Griffiths RR , Richards WA , Johnson MW , McCann UD , Jesse R. Mystical-type experiences occasioned by psilocybin mediate the attribution of personal meaning and spiritual significance 14 months later. Journal of Psychopharmacology 2008;22(6):621–321859373510.1177/0269881108094300PMC3050654.

27.

Barrett FS , Johnson MW , Griffiths RR . Validation of the revised Mystical Experience Questionnaire in experimental sessions with psilocybin. Journal of Psychopharmacology 2015;29(11):1182–902644295710.1177/0269881115609019PMC5203697.

28.

Vollenweider FX , Preller KH . Psychedelic drugs: Neurobiology and potential for treatment of psychiatric disorders. Nature Reviews Neuroscience 2020;21(11):611–243292926110.1038/s41583-020-0367-2.

29.

Carbonaro TM , Bradstreet MP , Barrett FS , MacLean KA , Jesse R , Johnson MW , et al. Survey study of challenging experiences after ingesting psilocybin mushrooms: Acute and enduring positive and negative consequences. Journal of Psychopharmacology 2016;30(12):1268–782757876710.1177/0269881116662634PMC5551678.

30. See note 11, Griffiths et al. 2006, at 268–83.

31. See note 12, Griffiths et al. 2011, at 649–65.

32. See note 11, Griffiths et al. 2006, at 268–83.

33. See note 29, Carbonaro et al. 2016, at 1268–78.

34. See note 29, Carbonaro et al. 2016, at 1268–78.

35.

Studerus E , Kometer M , Hasler F , Vollenweider FX . Acute, subacute and long-term subjective effects of psilocybin in healthy humans: A pooled analysis of experimental studies. Journal of Psychopharmacology 2011;25(11):1434–522085534910.1177/0269881110382466.

36.

Nutt DJ , King LA , Phillips LD . Drug harms in the UK: A multicriteria decision analysis. The Lancet 2010;376(9752):1558–6510.1016/S0140-6736(10)61462-621036393.

37. See note 11, Griffiths et al. 2006, at 268–83.

38. See note 12, Griffiths et al. 2011, at 649–65.

39. See note 13, Griffiths et al. 2018, at 49–69.

40. See note 14, Griffiths et al. 2016, at 1181–97.

41. See note 11, Griffiths et al. 2006, at 268–83.

42. See note 12, Griffiths et al. 2011, at 649–65.

43. See note 12, Griffiths et al. 2011, at 649–65.

44. See note 13, Griffiths et al. 2018, at 49–69.

45. See note 14, Griffiths et al. 2016, at 1181–97.

46. See note 26, Griffiths et al. 2008, at 621–32.

47.

Yaden DB , Yaden ME , Griffiths RR . Psychedelics in psychiatry—Keeping the renaissance from going off the rails. JAMA Psychiatry 2021;78(5):469–703326372010.1001/jamapsychiatry.2020.3672PMC8102315.

48. See note 1, Olson 2020, at 563–7.

49. See note 1, Olson 2020, at 563–7.

50.

Charon R. Narrative medicine: A model for empathy, reflection, profession, and trust. JAMA 2001;286(15):1897–9021159729510.1001/jama.286.15.1897.

51. See note 7, Johnson et al. 2008, at 603–20.

52. World Health Organization. Preamble to the Constitution of the World Health Organization as adopted by the International Health Conference, New York, 19–22 June, 1946; signed on 22 July 1946 by the representatives of 61 States (Official Records of the World Health Organization, no. 2, p. 100) and entered into force on 7 April 1948.

53.

Beauchamp TL , Childress JF . Principles of Biomedical Ethics. Oxford: Oxford University Press; 2001 .

54. See note 2, Yaden, Griffiths 2020, at 568–72.

